# Semantically Enriched Data Access Policies in eHealth

**DOI:** 10.1007/s10916-016-0581-7

**Published:** 2016-09-24

**Authors:** Michał Drozdowicz, Maria Ganzha, Marcin Paprzycki

**Affiliations:** 1 Systems Research Institute Polish Academy of Sciences, Warsaw, Poland; 2 Department of Mathematics and Information Sciences, Technical University of Warsaw, Warsaw, Poland

**Keywords:** IoT, XACML, Ontologies, Semantic technologies, Access management, eHealth, HL7

## Abstract

Internet of Things (IoT) requires novel solutions to facilitate autonomous, though controlled, resource access. Access policies have to facilitate interactions between heterogeneous entities (devices and humans). Here, we focus our attention on access control in eHealth. We propose an approach based on enriching policies, based on well-known and widely-used eXtensible Access Control Markup Language, with semantics. In the paper we describe an implementation of a Policy Information Point integrated with the *HL7 Security and Privacy Ontology*.

## Introduction

Rising number of connected devices, including networks of sensors, opens questions related to data privacy and security; on all levels of the ecosystem. Regulation of access to data and services, exposed by the “components” of the IoT, is similar to that of the Web resources and services. There is an “entity”, described with attributes, or roles that requests access to data and/or resource(s) (or services available within resources). In response, based on declarative or imperative rules, such request is granted (or denied). In the IoT, the core use-case involves interacting devices (rather than human-computer interactions). Furthermore, simple attribute or role-based approaches to access control, may not work. The main reasons are: (i) huge number of resources ([2, 12]), (ii) fast growing number of consumers ([13, 15, 24]), (iii) heterogeneity of data and services ([10, 11, 14, 16]), (iv) dynamics of (often short-lived) interactions ([18, 19, 23]).

Data privacy, and thus access control, is particularly important in eHealth. Here, the Internet of Things brings about a number of potential benefits, such as constant monitoring of patient’s medical parameters, online consultations, or remote control of medical devices (e.g. insulin pumps). It also increases the need for access control as, in the IoT scenarios, available patient data becomes even more detailed, making it extremely sensitive. Furthermore, personal data, of a single patient, may be geographically distributed (e.g. across patient’s home, multiple medical institutions and devices, etc.).

Here, we discuss a semantically enriched access control policy system. In comparison to our previous publications on the topic, here we present how the solution has been integrated with an existing eHealth privacy ontology and provide a relevant example of benefits that such an approach provides.

To this effect, we start by introducing the state-of-the-art, in the area of interest, in “State-of-the-art”. In “Privacy and access control in eHealth”, we describe the background for employing policy-based access control in the field of eHealth, as well as the unique challenges arising when it is combined with the Internet of Things technologies. “XACML”, provides a brief introduction to the XACML policy language that we have selected as the basis of our work. We follow, in “HL7 security and privacy ontology”, with an introduction to the *HL7 Security and Privacy Ontology*. Having provided the details of the work we have built upon, in “Semantic policy information point”, we describe the architecture of the Semantic Policy Information Point. “Example of enforcing access control” gives an example of using the semantic extensions to the XACML, while evaluating a data access request against a policy defined in the system. We follow with an outline of the proposed approach to the (re)design of the *Policy Information Point*.

## State-of-the-art

Initial study of pertinent literature has been included in the conference version of this paper. There, we have discussed state-of-the-art concerning: (a) policy based access control, (b) semantic approaches to access control policies, and (c) semantic extensions to the XACML (for a complete presentation see, [22]). Therefore, here we focus our attention on the remaining / newly found reference-points that jointly provide context for our present work.

There have been attempts at implementing policy based access control in the context of access control in the domain of eHealth and pervasive healthcare. In [27], a framework for context-aware Role Based Access Control, that utilizes XACML for policy specification and enforcement together with an ontology is proposed. In this solution, a semantic reasoner is used for dynamic role assignment, depending on the access control request context (both domain-specific and environmental). With respect to this research, our approach makes broader use of semantic inference – providing not only additional subject role specification, but also enriching the general attribute space of the decision context, thus focusing more on the Attribute Based Access Control model.

In [26], a similar approach is suggested, bringing more attention to the problem of heterogeneity and interoperability between systems using different vocabularies. While the solution provides great flexibility in defining rules and mapping concepts to a common ontology, in our opinion, its reliance on a custom policy engine results in limited robustness and practical applicability.

One important finding from our discussions with colleagues outside of the academia is that, while standards slowly find their way to EHR systems, providing greater uniformity across solutions and easier integration between institutions, access control is typically handled in a proprietary way. In this context, our proposal to build on top of the XACML as the policy language and standard ontologies, such as the *HL7 Security and Privacy Ontology*, may contribute to seeing wider adoption of more general / interoperable methods.

## Privacy and access control in eHealth

It should be obvious that eHealth solutions, placed within the IoT, require better managed data access. Currently, patients’ medical records are usually protected only through a coarse-grained access control – based on simple lists of system users, perhaps extended with a role-permission model. However, when looking at the medical information, it is possible to distinguish different levels of data confidentiality and sensitivity. For example, the HL7 standard defines 6 levels of confidentiality [6] and the following sensitivity categories [5]:

- *ETH* – substance abuse information sensitivity
- *GDIS* – genetic disease information sensitivity
- *HIV* – HIV/AIDS information sensitivity
- *PSY* – psychiatry information sensitivity
- *SDV* – sexual assault, abuse, or domestic violence information sensitivity
- *SEX* – sexuality and reproductive health information sensitivity
- *SICKLE* – sickle cell
- *STD* – sexually transmitted disease information sensitivity
- *TBOO* – taboo

On the other hand, note that the information about body parameters (blood pressure, temperature etc.) can (and should) be accessible to multiple “personnel roles”, e.g. nurses, physiotherapists, etc.

A typical example, where finer grained access control could provide better privacy, involves a hospital, where (typically) every physician has full access to the medical history of every patient. However, such access is not necessary. It would suffice if the physician had access to the information about her patients, with some additional privileges granted during her “work shifts” (when she would need access to data of “all patients”). Similarly, access to data generated by (“external”) devices, gathering information about the patient (or, more importantly, controlling treatment parameters), should be restricted to the explicitly defined personnel.

Another interesting question, concerning access control in “IoT for eHealth”, is: where access authorization takes place. Obviously, if all data is curated by a single institution, access policies are enforced there. With the IoT, however, most sensors are accessible via a gateway (not directly), responsible for securing the connection and, sometimes, also for aggregating and storing the acquired information. Consequently, it becomes possible to provide an additional authorization point within the gateway, which could be a part of the patient’s “own infrastructure”. Such solution (i) makes data access management more complex for the medical institution, but (ii) gives more control to the owner of the data, and (iii) may be valuable when the patient is treated in multiple institutions (by multiple doctors). Here, access control may be based on patient-doctor relation, rather than associated with specific institutions.

## XACML

Keeping this in mind, let us now briefly describe one of most popular approaches to managing data access. The eXtensible Access Control Markup Language (XACML; [4]) is a declarative language for specifying Attribute Based Access Control (ABAC; [25]) policies. It uses the XML as the internal format, but many implementations handle information transfer in other formats, e.g. the SAML ([1]). For a more detailed description of the language and analysis of benefits and drawbacks of using it to specify policy rules we refer to [22]. Here, we will focus on the reference architecture of an XACML processing system, to provide the necessary background needed to understand how our extension, described in further sections, fits into it.

To make the following description of the XACML engine more comprehensible let us assume that we are dealing with a system supporting work of a hospital. The system stores the Electronic Health Record (EHR) data for all its patients. Furthermore it integrates with a number of connected devices, providing remote health monitoring facilities. Obviously, there are many users of the system and rules regulating, which users have access to which parts of patients’ data. The XACML provides the possibility to manage and enforce these rules (outside of the services that actually provide the functionality for the users) and enables easy administration and modifications of the policies.

A typical XACML engine consists of the following major components:

- *Policy Enforcement Point* (PEP) – responsible for the actual act of enabling or preventing access to the resource (e.g. to patient’s blood pressure record). It also coordinates the execution of, so called, *Obligations*, which are additional operations that should be performed when a decision has been made (e.g. logging the request for auditing purposes).
- *Policy Information Point* (PIP) – a source of attribute values (e.g. specifying that Nick Riviera is a doctor, while Jean Bloom is a patient).
- *Context Handler* – which converts requests and responses between native formats and the XACML canonical representation and coordinates, with the PIPs, gathering of the required attribute values (e.g. checking who is Nick Riviera, asking to access blood pressure data).
- *Policy Decision Point* (PDP) – which evaluates policies and issues the final authorization decisions (e.g. establishes that Nick Riviera as a doctor has the right to access the blood pressure data of Jean Bloom).
- *Policy Administration Point* (PAP) – which defines, stores and manages policies.

**Fig. 1:**
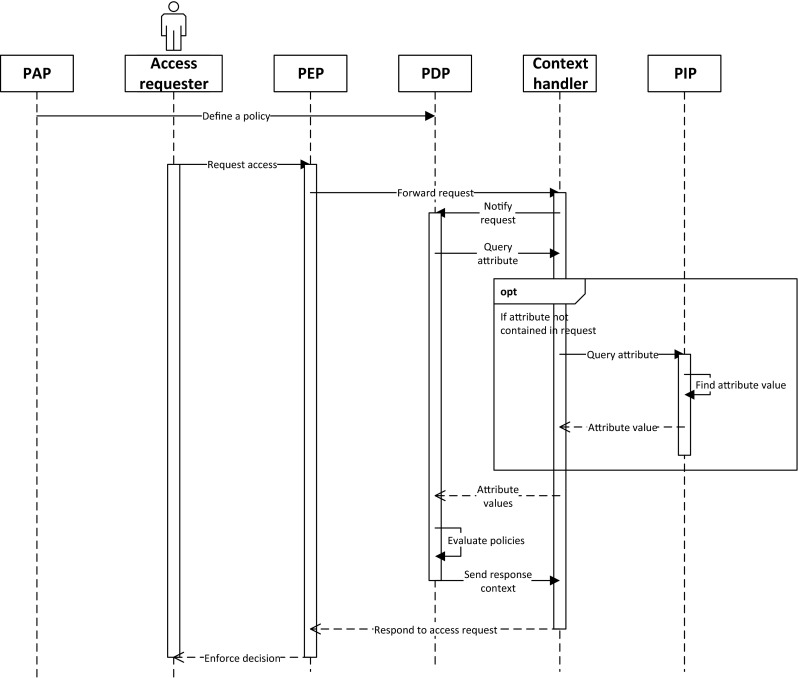
Evaluation of an access request in a standard XACML architecture

Having described the XACML reference architecture, in Fig. 1, we depict the sequence of messages in an access control granting process. Before the system is used, an administrator defines policies within the *PAP* and supplies them to the *PDP*. When a user (e.g. Nick Riviera) wishes to access protected, data (of Jean Bloom), the medical system (Acess Requester) communicates with the *PEP*. The request is forwarded to the *Context Handler*, which in turn notifies the *PDP* of the request. Next, the *PDP* starts evaluating the request against policies, first, retrieving values of all attributes used in policy definitions. These might reflect the identity and role/group information of the user, some properties describing the patient, or the type of the requested EHR information (e.g. finding that Nick Riviera is a doctor, while Jean Bloom is a patient). If the attribute value has been supplied in the request, the *Context Handler* returns it directly. Otherwise, it queries the *PIP*, which can be an EHR store or other data source. When the *PDP* has all the required information, it evaluates the policy rules, combines the results (if multiple policies are in effect) and builds the response context, which is returned to the *Context Handler*. The result of the evaluation are sent to the *PEP*, which enforces the decision (e.g. granting access to the medical data) and may also execute any obligations specified in the policy (e.g. record the event in a security journal).

While a very powerful tool, the XACML lacks possibilities to (i) *reason about the domain* containing the attributes, and (ii) *infer additional data* in an automatic way. Moreover, the XACML standard deals only with the policy definition and enforcement, while it does not provide solutions for the attribute management. To be able to deal with these shortcomings, we propose to introduce semantic data processing. Therefore, let us introduce a pertinent ontology that will allow us to introduce and illustrate the proposed approach.

## HL7 security and privacy ontology

In [7], we read that *The HL7 Security and Privacy Ontology serves to name, define, formally describe, and interrelate key security and privacy concepts within the scope of Healthcare Information Technology (Healthcare IT)*. This ontology is expressed in OWL, and can be used by itself to provide access control decisions by means of classifying a request using a semantic reasoner. As such, it can be classified as a fully semantic approach to access control policies. However, the ontology can be also used in conjunction with another policy specification language, such as the XACML.

We have found that separating the policy definitions from the domain concepts, reflected in an ontology, provides a more maintainable and expressive solution than defining the policies solely as ontological concepts (as possible through the HL7 ontology). The reasons for that include:

- XACML is widely used in the industry and has very good tool support. As such, it is easier to extend existing XACML systems with a semantic component than it would be to create custom decision points working solely with ontological policies (note that purely semantic approaches have failed in the past, see [22]).
- Combining policies in the HL7 ontology is made by using logical operators allowing to decide whether the request is an instance of a policy or not. In comparison, the XACML standard provides multiple built-in policy combining algorithms and an extension point to define new ones. This solution makes it much easier to express rules such as Deny-overrides or Permit-overrides.
- The datatype restrictions in OWL2 are limited to the XSD facets. The XACML, on the other hand, provides a wider range of functions that can be used for comparing restrictions in the policy with the values of request attributes. It also provides a mechanism for extending the language with custom functions.

**Fig. 2:**
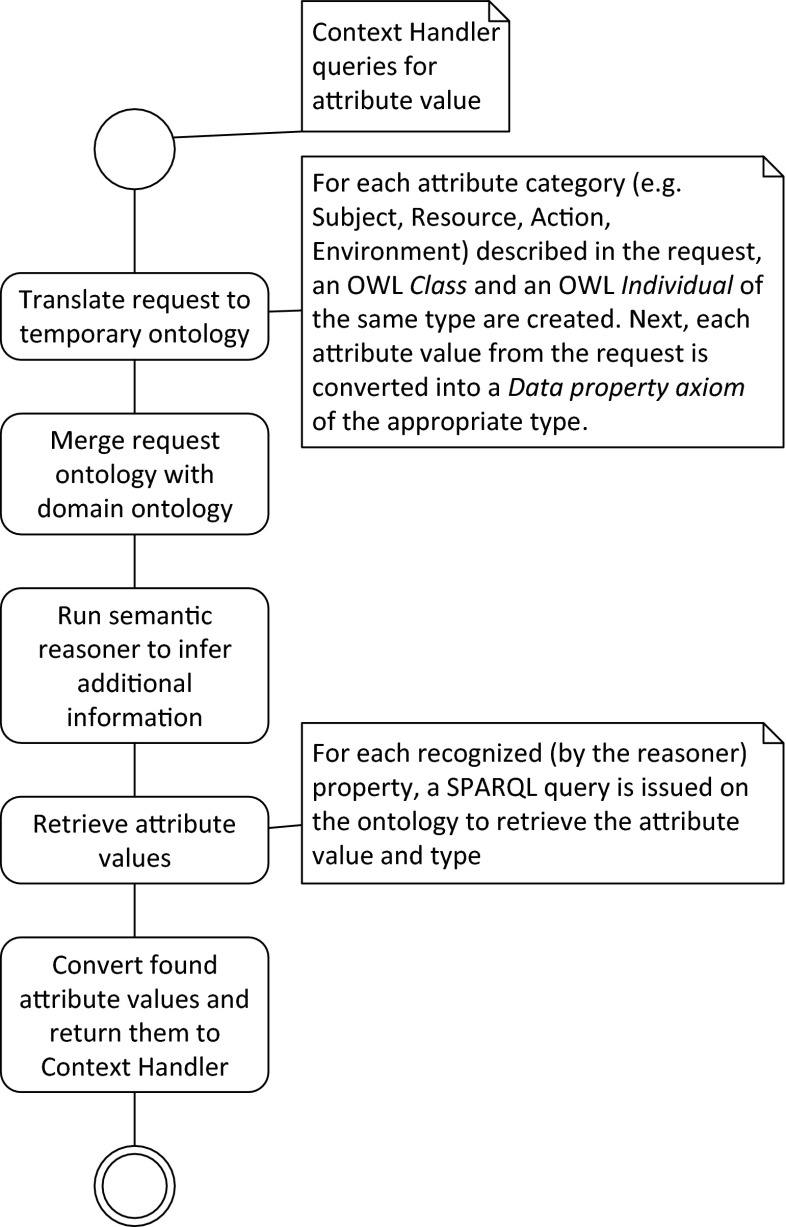
Algorithm for providing required attribute values

Therefore, we have considered if it would be possible to combine the HL7 Security and Privacy Ontology with the XACML-based access control. The remaining parts of this paper discuss how we have actually achieved this goal.

## Semantic policy information point

As outlined in [22], following [28], we have proposed a different *Semantic Policy Information Point* (SemanticPIP), for the XACML reference architecture. The proposed SemanticPIP is capable of providing values to unknown attributes, *by inferring them* from the ontologies describing the domain of the system. The general algorithm used by the SemanticPIP is presented in Fig. 2.

Listing 1, shows a sample SPARQL query used for selecting the attribute. Here, the categoryId parameter is replaced, during the runtime, with the unique identifier of the *Individual* created in Step 1 – querying Context Handler. For instance, the identifier may represent the user accessing the resource, or the resource under evaluation. Further, the attributeId is a fully qualified *id* of the requested attribute (e.g. type of information being accessed).

**Listing 1:**
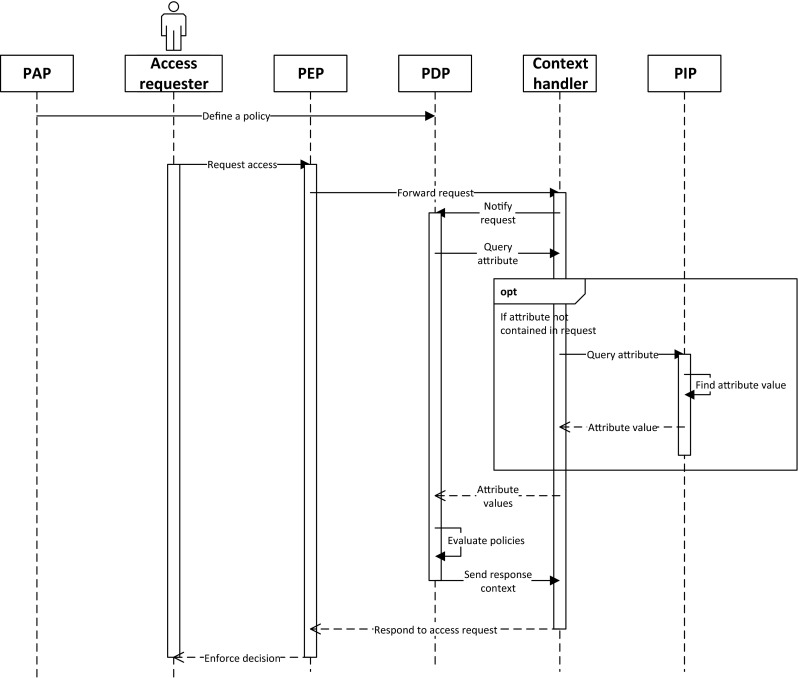
SPARQL query

It should be stressed that, compared with [28], our solution *does not change* the reference architecture of the XACML system. Instead, it implements the contract of the PIP component. Furthermore, the SemanticPIP queries only for, and returns, the attribute values that are requested by the *Context Handler*, reducing the burden placed on the SPARQL engine.

The component has been implemented as an attribute finder extension to the *Balana Framework* ([3, 17]), an XACML engine developed by the WSO2 as a continuation of the popular Sun’s XACML Implementation. The engine was chosen due to its maturity and widespread use, among others, as part of the WSO2 Identity Server.

## Example of enforcing access control

**Fig. 3:**
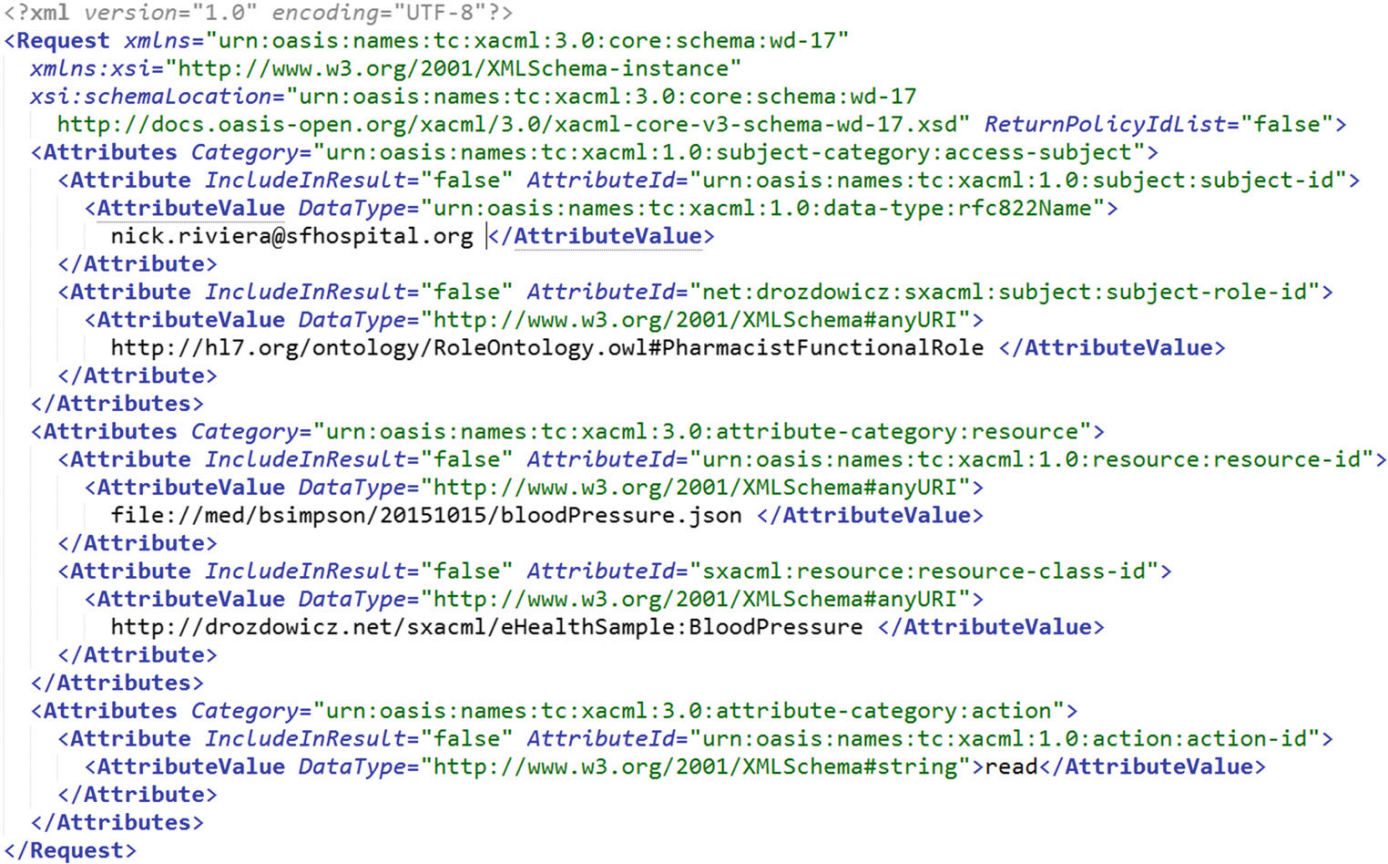
Example XACML request

**Fig. 4:**
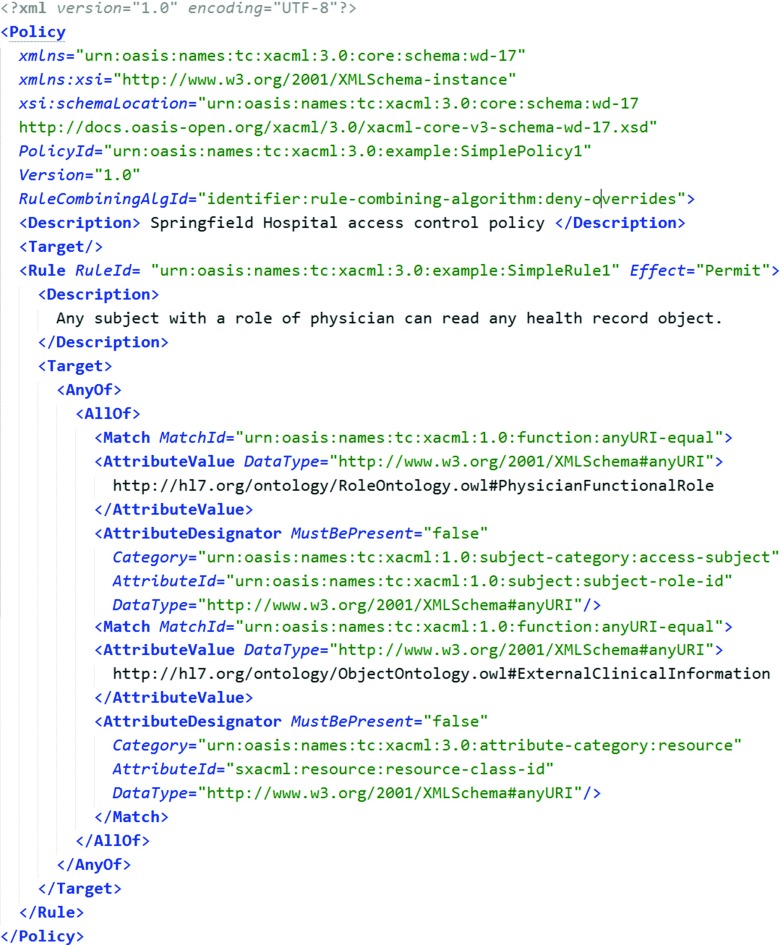
Example XACML policy

As an example of the approach, let us consider a scenario in which a blood pressure sensor is working in the patient’s (Jean Bloom) apartment. Let us assume that an external system, acting on behalf of a physician (Nick Riviera, authenticated as nick.riviera@sfhospital.org, in role http://hl7.org/ontology/RoleOntology.owl#PhysicianFunctionalRole), wishes to access the result of the observation (see, Fig. 3). The observation is identified by the *resource-id* of file://med/bsimpson/20151015/bloodPressure.json and the *resource-class-id* of http://drozdowicz.net/sxacml/eHealthSample:BloodPressure. There exists an XACML policy describing the rules for permitting access. However, as can be seen in Fig. 4, this policy does not cover *specifically* the blood pressure measurement. Instead, it states that a person in a role physician (http://hl7.org/ontology/RoleOntology.owl#PhysicianFunctionalRole) may access clinical observations of a patient (identified as *resource-class-id* of http://hl7.org/ontology/ObjectOntology.owl#ExternalClinicalInformation).

**Fig. 5:**
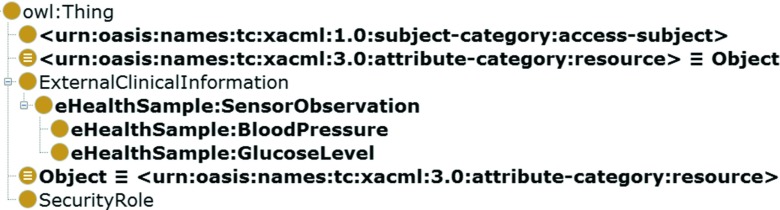
Ontology enriching the XACML policy

Apart from the policy, the authorization system also uses an ontology that describes relationships between needed concepts (see, Fig. 5). This ontology is based on the HL7 Privacy and Security Ontology, by using the Object class as an equivalent of the XACML resource.

Acting on the request, the XACML engine asks the SemanticPIP for the resource-class-id attribute value. The SemanticPIP uses the semantic reasoner to infer that the BloodPressure class is a subclass of the ExternalClinicalInformation (from the HL7 Security and Privacy ontology). This information is added to the request context and used by the XACML engine to permit the request.

**Listing 1:**
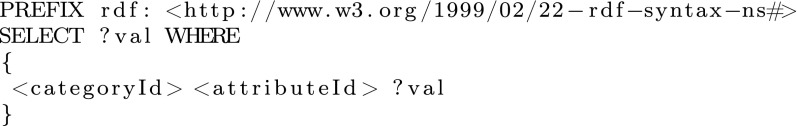
SPARQL query

Observe that, in this scenario, the system accessing the data needs not specify that it requests an ExternalClinicalInformation resource – it is not required to know the exact model of the domain on the provider’s side. As a matter of fact, using some ontology matching algorithms it would even be possible for the requestor and the owner to communicate using slightly different vocabularies, thus greatly simplifying the interoperation between heterogeneous systems. Moreover, in this example, we have demonstrated the ability to import existing ontologies, namely the RoleOntology and the ObjectOntology, parts of the HL7 Security and Privacy ontology, that already provide concepts describing the domain.

## Conclusions and future work

The aim of this paper was to further discuss issues involved in instantiating rule-based resource access policies in the IoT ecosystem. The proposed Semantic Policy Information Point (SemanticPIP) has been implemented and is being thoroughly tested. There are two scenarios where it is going to be applied. First, a non-IoT one, originating from the *Agents in Grid* project (see, [20, 21]). The IoT application, on the other hand, will be focused on users that attend nutritional outpatient care centers. Here a body sensor network will be combined with measurements taking place in the medical facility, leading to a situation where complex data access patterns materialize. This will be realized within the scope of the Inter-IoT EU project. Finally, the SemanticPIP development plans include also support for the additional XACML profiles, namely the Role Based Access Control Profile ([8]) and the Hierarchical Resource Profile ([9]).
